# The effect of coenzyme Q10 supplementation on liver enzymes: A systematic review and meta‐analysis of randomized clinical trials

**DOI:** 10.1002/fsn3.3478

**Published:** 2023-06-07

**Authors:** Mohadeseh Soleimani Damaneh, Somaye Fatahi, Naheed Aryaeian, Hossein Bavi Behbahani

**Affiliations:** ^1^ Department of Nutrition, School of Public Health Iran University of Medical Sciences Tehran Iran; ^2^ Department of Clinical Nutrition and Dietetics, Faculty of Nutrition and Food Technology Shahid Beheshti University of Medical Sciences Tehran Iran; ^3^ Pediatric Gastroenterology, Hepatology, and Nutrition Research Center, Research Institute for Children's Health Shahid Beheshti University of Medical Sciences Tehran Iran; ^4^ Student Research Committee Ahvaz Jundishapur University of Medical Sciences Ahvaz Iran

**Keywords:** clinical trials, coenzyme Q10, liver enzymes, meta‐analysis

## Abstract

Coenzyme Q10 is a potent antioxidant and is necessary for energy production in mitochondria. Clinical data have suggested that coenzyme Q10 (CoQ10) has some beneficial effects on liver function. However, these results are equivocal. This systematic review and meta‐analysis aimed to clarify the effect of coenzyme Q10 supplementation on the serum concentration of liver function enzymes. We searched the online databases using relevant keywords up to April 2022. Randomized clinical trials (RCTs) investigating the effect of CoQ10, compared with a control group, on serum concentrations of liver enzymes were included. We found a significant reduction following supplementation with CoQ10 on serum concentrations of alanine aminotransferase (ALT) based on 15 effect sizes from 13 RCTs (weighted mean difference [WMD] = −5.33 IU/L; 95% CI: −10.63, −0.03; *p* = .04), aspartate aminotransferase (AST) based on 15 effect sizes from 13 RCTs (WMD = −4.91 IU/L; 95% CI: −9.35, −0.47; *p* = .03) and gamma‐glutamyl transferase (GGT) based on eight effect sizes from six RCTs (WMD = −8.07 IU/L; 95% CI: −12.82, −3.32; *p* = .001; *I* ^2^ = 91.6%). However, we found no significant effects of CoQ10 supplementation on alkaline phosphatase concentration (WMD = 1.10 IU/L; 95% CI: −5.98, 8.18; *p* = .76). CoQ10 supplementation significantly improves circulating ALT, AST, and GGT levels; therefore, it might positively affect liver function. Further high‐quality RCTs with more extended intervention periods and larger sample sizes are recommended to confirm our results.

## Graphical abstract

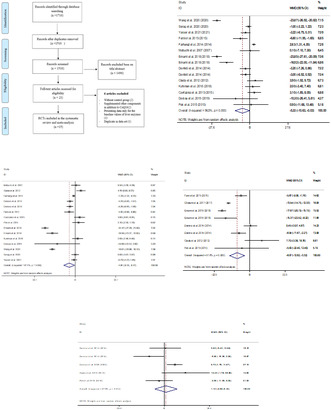

## 1 INTRODUCTION

Liver diseases and elevated liver enzymes are global health problems causing approximately two million deaths annually (Asrani et al., 2019). To screen for liver disorders, the most common liver function tests include alanine aminotransferase (ALT), aspartate aminotransferase (AST), alkaline phosphatase (ALP), and gamma‐glutamyl transferase (GGT). Transaminases (ALT and AST) have essential functions in the glucose and amino acid metabolism pathways by producing pyruvate and oxaloacetate in the body, especially in the liver (Wroblewski & Ladue, 1956a, 1956b). ALT and AST are found primarily in liver cells, but AST is also present in skeletal muscle, kidney, heart, brain, and red blood cells (Jastrzębski et al., 2015), and also the concentration of AST is lower than that of ALT in hepatocytes. ALT is therefore a more specific marker for liver disease than AST (Sampson et al., 1980; Weibrecht et al., 2010). ALP is a hydrolase enzyme that catalyzes the hydrolysis of pyrophosphate, which is an inhibitor of vascular calcification and leads to the facilitation of vascular calcification. Therefore, ALP plays an important role in increasing the risk of atherosclerosis. Serum ALP levels are commonly used to diagnose liver damage or bone disorders (Tonelli et al., 2009). GGT is an enzyme embedded in the outer surface of cell remembrance throughout the body, but it is mostly found in the liver. High levels of GGT in the blood have been regarded as a marker of liver disease, damage to the bile ducts, or alcohol consumption (Whitfield, 2001). GGT plays a significant role in the catabolism of glutathione (GSH). Glutathione is considered a major intracellular water‐soluble antioxidant. Therefore, GGT can increase oxidant stress in the body through its role in glutathione homeostasis.

Several studies have shown that damage to liver cells for some reasons, including inflammation, oxidative stress, or an increase in fat storage in the liver, can lead to elevated liver enzyme levels (Ahn et al., 2014; Bonnet et al., 2011; Suda et al., 2008; Zhang et al., 2010). These enzymes are also increased with muscle damage following intense exercise (Halonen & Konttinen, 1962). Epidemiological studies have indicated significant relationships between these enzymes and type 2 diabetes mellitus (Fraser et al., 2009; Kunutsor et al., 2013), cardiovascular disease (Fraser et al., 2007; Kim et al., 2005; Schindhelm et al., 2007), and mortality from vascular and nonvascular diseases (Hyeon et al., 2004; Ruttmann et al., 2005; Tonelli et al., 2009). Although different conditions and factors can cause the elevation of liver enzymes, evaluation and treatment should be based on identifying and eliminating the primary cause. However, many studies have shown the effectiveness of nondrug treatments and dietary supplements.

Coenzyme Q10 is a vitamin‐like compound that is distributed in two reduced (ubiquinol‐10) and an oxidized form (ubiquinone‐10) in the human body (Desbats et al., 2015). CoQ10 plays an essential role in the respiratory chain as an electron carrier in mitochondrial ATP synthesis (Arenas‐Jal et al., 2020). CoQ10 is a potent anti‐inflammatory agent which can reduce the production of proinflammatory cytokines (Bentinger et al., 2010; Schmelzer et al., 2009). In addition, CoQ10 acts as a powerful antioxidant and protects cells by disrupting lipid peroxidation (Forsmark‐Andree & Ernster, 1994). Moreover, indirectly plays as an antioxidant by regenerating other antioxidants such as α‐tocopherol and ascorbate (Marcoff & Thompson, 2007). Considering that free radicals and lipid peroxidation can reduce antioxidant levels and thus make hepatocytes susceptible oxidative damage (Li et al., 2014), CoQ10 supplementation as an antioxidant compound with anti‐inflammatory effects, and by stabilizing and regenerating the body's antioxidant defense system, may be effective in a variety of diseases, including NAFLD, and other diseases that lead to elevated enzyme levels (Mantle & Preedy, 1999; Spahis et al., 2017). Several animal and human studies have shown that supplementation with CoQ10 has been effective in protecting the liver and in conditions that lead to liver disorders and consequently elevated liver enzymes level (Celik et al., 2006; Leelarungrayub et al., 2010; Sumimoto et al., 1987). At the same time, other studies have not shown such an effect. Due to the contradictions in the results of various studies regarding the effectiveness of CoQ10 supplementation on the level of liver enzymes, this systematic review and meta‐analysis study was conducted to estimate the overall effect of CoQ10 supplementation on liver enzymes in RCT studies.

## 2 METHODS

This study was performed based on the Preferred Reporting Items for Systematic Reviews and Meta‐Analyses (PRISMA) protocol for reporting systematic reviews and meta‐analyses.

### 2.1 Search strategy

We performed a comprehensive literature search in the online databases of PubMed, Scopus, Web of Science, and Google Scholar up to April 2022. In the search, Medical Subject Heading (MeSH) terms and non‐MeSH terms were applied to evaluate the impact of coenzyme Q10 supplementation on Liver Enzymes. The following keywords were used in the search strategy: (“Coenzyme Q10” OR “Q10” OR “CoQ10” OR “Ubiquinone” OR “Ubidecarenone” OR “Bio‐Quinone Q10” OR “co‐enzyme Q10” OR “Ubiquinol‐10” OR “ubiquinone‐10”) AND (“Liver enzyme*” OR “Liver function test*” OR “Alanine transaminase” OR “SGPT” OR “Serum Glutamic‐Pyruvic transaminase” OR “Glutamic alanine transaminase” OR “Alanine aminotransferase” OR “Alanine aminotransferase” OR “ALT” OR “Aspartate aminotransferase” OR “Aspartate transaminase” OR “SGOT” OR “Serum Glutamic Oxaloacetic transaminase” OR “AST” OR “Alkaline Phosphatase” OR “ALP” OR “Gamma‐Glutamyltransferase” OR “γ‐glutamyltransferase” OR “GGT”). No language or time restriction was applied. Reference lists of the relevant studies were manually screened to avoid missing any eligible publications. Unpublished studies were not considered. The literature search was conducted by two independent investigators.

### 2.2 Inclusion criteria

We included eligible studies that met the following criteria: (1) randomized controlled clinical trials, (2) studies that administered coenzyme Q10 supplementation in different chemical forms, (3) RCTs with at least 1 week's duration of intervention, and (4) controlled trials that reported mean changes and their standard deviations (SDs) of liver enzymes throughout the trial for both intervention and control groups or presented required information for calculation of those effect sizes. If >1 article was published for one dataset, the more complete one was included. Clinical trials with an additional intervention group were considered two separate studies.

### 2.3 Exclusion criteria

In the current meta‐analysis, we excluded studies with a cohort, cross‐sectional, and case–control design, review articles, and ecological studies. We also excluded trials without a control group.

### 2.4 Data extraction

Two independent investigators performed data extraction from each eligible RCT. The following information was extracted: name of the first author, publication year, individuals' characteristics (mean age, BMI, and sex), design, sample size (control and intervention groups), the dosage of coenzyme Q10, duration of intervention, and mean changes and their SDs of liver enzymes throughout the trial for the intervention and control groups. If data on liver enzymes were reported in different units, we converted them to the most frequently used unit.

### 2.5 Risk of bias assessment

We applied the Cochrane quality assessment tool to assess the risk of bias for each study included in the current meta‐analysis (Higgins et al., 2011). This tool contained seven domains including, random sequence generation, allocation concealment, reporting bias, performance bias, detection bias, attrition bias, and other sources of bias. Each domain was given a “high risk” score if the study comprised methodological defects that may have affected its findings, a “low risk” score if there was no defect for that domain, and an “unclear risk” score if the information was not sufficient to determine the impact. If the trial had “low risk” for all domains, it was considered a high‐quality study with a totally low risk of bias. The risk of bias assessment was done independently by two reviewers.

### 2.6 Statistical analysis

Mean changes and their SDs of liver enzymes in the coenzyme Q10 and control groups were used to obtain the overall effect sizes. When mean changes were not reported, we calculated them by considering changes in liver enzymes during the intervention. We also converted standard errors (SEs), 95% confidence intervals (CIs), and interquartile ranges (IQRs) to SDs using the method of Hozo et al. (2005). To obtain the overall effect sizes, we applied a random‐effects model that takes between‐study variations into account. Heterogeneity was determined by the *I* ^2^ statistic and Cochrane's *Q* test. *I* ^2^ value >50% or *p* < .05 for the *Q*‐test was considered significant between‐study heterogeneity (Brondani et al., 2014; Zahedi et al., 2018). To find probable sources of heterogeneity, subgroup analyses were performed according to the predefined variables including duration of the intervention (>8 vs. ≤8 weeks), the dosage of coenzyme Q10 (≤200 vs. >200 mg/day), participants' health condition (CHD patients vs. type 2 diabetes vs. type 1 diabetes mellitus vs. nonalcoholic fatty liver disease vs. dyslipidemia patients vs. athletes), Age (≤19 years vs. 19–50 years vs. > 50‐year), BMI (normal weight vs. overweight vs. obese vs. NR). Sensitivity analysis was used to detect the dependency of the overall effect size on a particular study. The possibility of publication bias was examined by the formal test Egger. The meta‐analysis was carried out by using the Stata, version 11.2 (StataCorp). A *p*‐value <.05 was considered a significant level.

## 3 RESULTS

**FIGURE 1:**
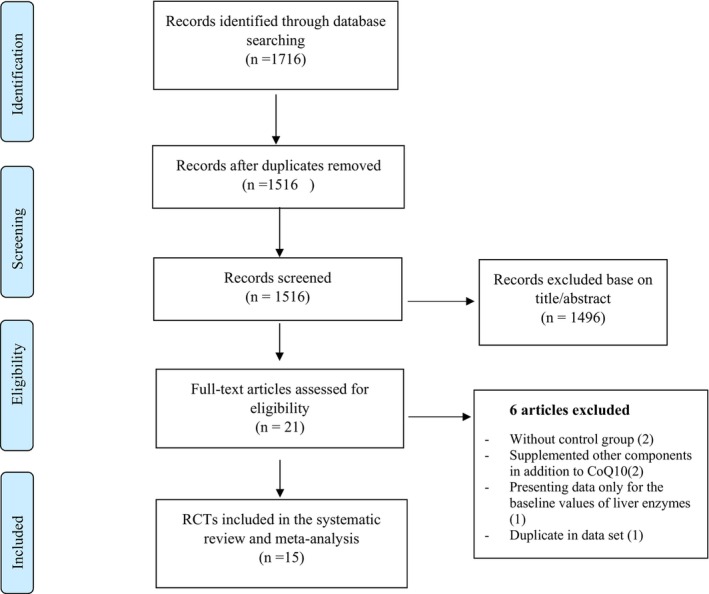
Flow diagram of study selection. RCTs, randomized clinical trials.

Of 1716 publications identified in our initial search, 200 duplicate articles were excluded. After screening the remaining 1516 records, 1496 unrelated articles were also rebased sis on the title and abstract assessment. Then, 21 publications remained for further evaluation of the full text. Of those 21 studies, two RCTs were excluded because they did not have a control group (Onur et al., 2014; Shafieipour et al., 2018). The study of Liu et al. (2016) was also excluded because they did not report changes in the levels of liver enzyme concentrations making us unable to calculate the mean changes in liver enzymes throughout the trial. We also excluded the two studies in which the effects of coenzyme Q10 in combination with other components were investigated (Curcio et al., 2020; Tóth et al., 2017). Moreover, two eligible articles were published on the same dataset (Farhangi et al., 2014; Jafarvand et al., 2016), of which the more complete one was included (Farhangi et al., 2014), and the other one was excluded (Jafarvand et al., 2016). After these exclusions, 15 eligible RCTs remained for inclusion in the current systematic review and meta‐analysis (Derosa et al., 2019; Diaz‐Castro et al., 2020; Emami & Bazargani‐Gilani, 2018; Farhangi et al., 2014; Farsi et al., 2016; Gholami et al., 2019; Hernández‐Ojeda et al., 2012; Kuhlman et al., 2019; Mabuchi et al., 2007; Nevzat, 2015; Pek et al., 2016; Rodríguez‐Carrizalez et al., 2016; Serag et al., 2020; Wang et al., 2020; Yasser et al., 2021); Of these, 13 studies assessed serum concentrations of ALT and AST (Derosa et al., 2019; Emami & Bazargani‐Gilani, 2018; Farhangi et al., 2014; Farsi et al., 2016; Kuhlman et al., 2019; Mabuchi et al., 2007; Nevzat, 2015; Pek et al., 2016; Rodríguez‐Carrizalez et al., 2016; Serag et al., 2020; Wang et al., 2020; Yasser et al., 2021), six studies examined serum concentrations of GGT (Emami & Bazargani‐Gilani, 2018; Farsi et al., 2016; Gholami et al., 2019; Hernández‐Ojeda et al., 2012; Nevzat, 2015; Pek et al., 2016), and four studies evaluated serum concentration of ALP following coenzyme Q10 supplementation (Diaz‐Castro et al., 2020; Hernández‐Ojeda et al., 2012; Nevzat, 2015; Pek et al., 2016). The study conducted by Nevzat et al. (2015) compared the effects of two doses of coenzyme Q10 supplementation (100 and 200 mg/day) with placed in their study, and also for Emami and Bazargani‐Gilani (2018) study that divided participants into four groups (supplement only, supplement with precooling strategy, and control groups), we extracted two effect sizes from these studies and included them in our meta‐analysis as two separate studies. The flow diagram of study selection is outlined in Figure 1.

### 3.1 Characteristics of the included studies

The characteristics of 15 RCTs in the current systematic review and meta‐analysis are illustrated in Table 1. These RCTs were published between 2007 and 2021 and were from Asia (Emami & Bazargani‐Gilani, 2018; Farhangi et al., 2014; Farsi et al., 2016; Gholami et al., 2019; Mabuchi et al., 2007; Nevzat, 2015; Pek et al., 2016; Wang et al., 2020; Yasser et al., 2021), Europe (Derosa et al., 2019; Diaz‐Castro et al., 2020; Kuhlman et al., 2019), Mexico (Hernández‐Ojeda et al., 2012; Rodríguez‐Carrizalez et al., 2016), and Egypt (Serag et al., 2020). Three studies were exclusively performed on male subjects (Diaz‐Castro et al., 2020; Emami & Bazargani‐Gilani, 2018; Nevzat, 2015), one study on females (Gholami et al., 2019), and others on both genders. The sample size of included RCTs varied from 10 to 100 participants, resulting in a total sample size of 712 individuals. The mean age of participants was between 11 and 61 years. The dosage of coenzyme Q10 supplements varied from 100 to 400 mg/day, and the duration of intervention ranged from 2 to 24 weeks across selected RCTs. All studies employed a parallel design. Concerning the type of coenzyme Q10, one study administered ubiquinol (Pek et al., 2016), one study liquid CoQ10 (Derosa et al., 2019), and others performed the intervention with coenzyme Q10 supplements. In one trial, a maximal exercise program for 2 h every day (Nevzat, 2015), and in another one precooling strategy in elite swimmers (Emami & Bazargani‐Gilani, 2018) was applied to the groups in addition to the main intervention. RCTs were performed on CHD patients (Wang et al., 2020), patients with type 2 diabetes (Gholami et al., 2019; Hernández‐Ojeda et al., 2012; Rodríguez‐Carrizalez et al., 2016), patients with type 1 diabetes mellitus (Serag et al., 2020), nonalcoholic fatty liver disease (Farhangi et al., 2014; Farsi et al., 2016), dyslipidemia patients (Derosa et al., 2019; Kuhlman et al., 2019; Mabuchi et al., 2007; Pek et al., 2016; Yasser et al., 2021), and athletes (Diaz‐Castro et al., 2020; Emami & Bazargani‐Gilani, 2018; Nevzat, 2015). None of the RCTs had a low risk of bias in all domains of the Cochrane Risk of Bias Assessment tool (Table S1).

**TABLE 1:** Summary of clinical trials on the effect of CoQ10 supplementation on liver enzymes.

	Author/year/country	Design	Participants, *N*	Health condition	Age, year^a^	BMI	Intervention		Duration/week	Outcomes^b^			
							Treatment	Control		Treatment		Control	
										Baseline	Changes	Baseline	Changes
1	Farsi et al. (2016)/Iran	RA/PA/DB	M/F: 41 Int: 20, Con: 21	NAFLD	Int: 19–54 year Con: 19–54 year	Int: 28.23 ± 3.60 Con: 29.69 ± 5.76	CoQ10 100 mg	Placebo (starch)	12	ALT: 30.4 ± 9.5 AST: 39.6 ± 11.9 GGT: 32.4 ± 9.2	ALT: −6.95 ± 7.57 AST: −5.47 ± 6.18 GGT: −6.31 ± 5.74	ALT: 30.9 ± 11.2 AST: 40.5 ± 15.1 GGT: 33.7 ± 10.0	ALT: −0.15 ± 9.2 AST: −0.62 ± 6.73 GGT: −0.64 ± 9.2
2	Farhangi et al. (2014)/Iran	RA/PA/DB	M/F: 41 Int: 20, Con: 21	NAFLD	Int: 42.73 ± 10.77 Con: 42.18 ± 10.8	Int: 30.59 ± 3.98 Con: 28.75 ± 4.02	CoQ10 100 mg	Placebo	4	ALT: 35.7 ± 4.7 AST: 32.3 ± 2.6	ALT: −3.85 ± 3.06 AST: −5.47 ± 6.18	ALT: 28.8 ± 2.7 AST: 32.6 ± 2.9	ALT: −6.68 ± 1.66 AST: −0.62 ± 6.73
3	Wang et al. (2020)/China	RA/PA/	M/F: 84 Int: 42, Con: 42	CHD patients	Int: 56.74 ± 7.98 Con: 59.25 ± 8.76	Int: 22.08 ± 0.57 Con: 22.15 ± 0.54	CoQ10 150 mg + atorvastatin + conventional medication	Atorvastatin + conventional medication	12	ALT: 24.0 ± 9.8 AST: 20.6 ± 7	ALT: 0.23 ± 6.03 AST: 1.01 ± 4.32	ALT: 18.5 ± 7.3 AST: 19.1 ± 7.4	ALT: 24.1 ± 8.07 AST: 19.62 ± 6.18
4	Serag et al. (2020)/Egypt	RA/PA/	M/F: 40 Int: 21, Con: 19	Type 1 diabetes mellitus	Int: 11.9 ± 3.1 SDS Con: 12.6 ± 2.9 SDS	Int: 0.76 ± 0.98 SDS Con: 0.64 ± 0.85 SDS	CoQ10 100 mg + routine treatment + insulin	Routine treatment + insulin	12	ALT: 12 ± 2.9 AST: 22 ± 7.4	ALT: −1 ± 1.78 AST: −2 ± 4.5	ALT: 12 ± 2.9 AST: 18 ± 2.9	ALT: 0 ± 4.64 AST: −2 ± 5.33
5	Yasser et al. (2021)/Iraq	RA/PA/	M/F: 39 Int: 20, Con: 19	Dyslipidemia patients	Int: 59.24 ± 5.57 Con: 58.11 ± 7.10	Int: 32.54 ± 4.66 Con: 31.59 ± 5.08	CoQ10 100 mg + routine treatment + atorvastatin	Routine treatment + atorvastatin	12	ALT: 27.1 ± 8.8 AST: 21.2 ± 6.5	ALT: −4.75 ± 5.31 AST: −1.05 ± 3.96	ALT: 25 ± 8.24 AST: 22.4 ± 6.3	ALT: −1.53 ± 5.9 AST: −0.26 ± 3.82
6	Mabuchi et al. (2007)/Japan	RA/PA/DB	M/F: 49 Int: 24, Con: 25	Dyslipidemia patients	Int: 61 ± 8 Con: 60 ± 8	Int: 23.3 ± 2.7 Con: 23.9 ± 3.4	CoQ10 100 mg + atorvastatin	Placebo + atorvastatin	12	ALT: 26 ± 19 AST: 25.6 ± 8.5	ALT: 4.9 ± 15.73 AST: 3.2 ± 8.36	ALT: 26 ± 12 AST: 25.2 ± 6.6	ALT: 4.8 ± 8.94 AST: 2.7 ± 4.49
7	Gholami et al. (2019)/Iran	RA/PA/DB	F: 70 Int: 35, Con: 35	Type 2 diabetes	Int: 52.97 ± 1.04 Con: 53.68 ± 1.14	Int: 29.30 ± 0.6 Con: 28.51 ± 0.52	CoQ10 100 mg + routine treatment	Placebo (cellulose acetate) + routine treatment	12	GGT: 32.02 ± 3.9	GGT: −10.32 ± 2.46	GGT: 32.03 ± 3.7	GGT: 3.22 ± 2.7
8	Emami and Bazargani‐Gilani (2018)/Iran	RA/PA	M: 18 Int: 9, Con: 9	Elite swimmers	Int: 18.60 ± 1.14 Con: 18.71 ± 1.11	Int: 22.67 ± 0.66 Con: 22.11 ± 1.65	CoQ10 300 mg + swimming training	Placebo (lactose) + swimming training	2	ALT: 24.1 ± 5.1 AST: 31.7 ± 4.3 GGT: 17.6 ± 4.3	ALT: −12.1 ± 4.12 AST: −14.11 ± 2.87 GGT: −9.74 ± 2.88	ALT: 20.3 ± 6.2 AST: 27.3 ± 5.2 GGT: 12.7 ± 4.0	ALT: 11.75 ± 4.02 AST: 10.36 ± 3.21 GGT: 7.93 ± 2.56
9	Emami and Bazargani‐Gilani (2018)/Iran	RA/PA	M: 18 Int: 9, Con: 9	Elite swimmers	Int: 18.40 ± 1.14 Con: 18.71 ± 1.11	Int: 22.45 ± 2.01 Con: 21.66 ± 1.45	CoQ10 300 mg + precooling strategy	Placebo (lactose) + precooling strategy	2	ALT: 25.9 ± 9.6 AST: 32.9 ± 9.6 GGT: 15.8 ± 9.3	ALT: −1.72 ± 6.83 AST: −3.29 ± 6.28 GGT: −4.01 ± 10.43	ALT: 27.3 ± 5.1 AST: 34.8 ± 4.3 GGT: 14.3 ± 4.3	ALT: 15.2 ± 3.73 AST: 15.27 ± 3.54 GGT: 11.36 ± 3.32
10	Demirci et al. (2015)/Turkey	RA/PA	M: 10 Int: 5, Con: 5	Skiing athletes	Int: 21.80 ± 0.73 Con: 21.60 ± 0.51	Int: NR Con: NR	CoQ10 100 mg + 2 h training	2 h training	2	ALT: 24.8 ± 4.4 AST: 33.4 ± 3.2 GGT: 12.6 ± 5.6 ALP: 28.0 ± 12.5	ALT: −1 ± 2.68 AST: −1.4 ± 1.92 GGT: −0.15 ± 3.54 ALP: 0.83 ± 8.06	ALT: 25.8 ± 6,0 AST: 33.4 ± 3.2 GGT: 13.3 ± 5.9 ALP: 28.6 ± 12.8	ALT: 2.2 ± 3.78 AST: 2.8 ± 2.3 GGT: −0.6 ± 3.59 ALP: 0.2 ± 8.13
11	Demirci et al. (2014)/Turkey	RA/PA	M: 10 Int: 5, Con: 5	Skiing athletes	Int: 21.60 ± 0.51 Con: 21.60 ± 0.51	Int: NR Con: NR	CoQ10 200 mg + 2 h training	2 h training	2	ALT: 24.2 ± 3,8 AST: 33.4 ± 3.2 GGT: 12.5 ± 5.5 ALP: 27.7 ± 12.4	ALT: −0.8 ± 2.39 AST: −1.6 ± 1.99 GGT: −7.54 ± 4.03 ALP: −9.32 ± 7.63	ALT: 25.8 ± 6,0 AST: 33.4 ± 3.2 GGT: 13.3 ± 5.9 ALP: 28.6 ± 12.8	ALT: 2.2 ± 3.78 AST: 2.66 ± 2.3 GGT: −0.6 ± 3.59 ALP: 0.2 ± 8.13
12	Diaz‐Castro et al. (2020)/Spain	RA/PA/DB	M: 100 Int: 50, Con: 50	Firemen	Int: 38.9 ± 1.4 Con: 38.2 ± 1.2	Int: 25.0 ± 0.4 Con: 25.0 ± 0.5	CoQ10 200 mg	Placebo	2	ALP: 61.2 ± 15.0	ALP: 3.99 ± 13.55	ALP: 63.5 ± 19.3	ALP: −2.74 ± 11.58
13	Hernández‐Ojeda et al. (2012)/Mexico	RA/PA/DB	M/F: 49 Int: 24, Con: 25	Type 2 diabetes mellitus with polyneuropathy	Int: 55.3 ± 8.4 Con: 57.0 ± 8.9	Int: 29.4 ± 7.3 Con: 29.3 ± 4.3	Ubiquinone 400 mg + antidiabetic therapy	Placebo + antidiabetic therapy	12	ALT: 27.2 ± 18.8 AST: 22.2 ± 8.6 GGT: 42.0 ± 22.7 ALP: 96.7 ± 36.3	ALT: 3.3 ± 13.5 AST: 3.8 ± 8.79 GGT: −1.8 ± 13.62 ALP: 2.7 ± 24.69	ALT: 21.5 ± 9.1 AST: 20.1 ± 5.5 GGT: 56.6 ± 40.0 ALP: 107.9 ± 45.2	ALT: −0.6 ± 5.53 AST: −0.9 ± 4.14 GGT: −9.5 ± 24.62 ALP: −10.7 ± 29.4
14	Kuhlman et al. (2019)/Denmark	RA/PA/DB	M/F: 35 Int: 18, Con: 17	In primary prevention	Int: 62 ± 1 Con: 64 ± 2	Int: 27.7 ± 0.6 Con: 28.8 ± 0.7	CoQ10 400 mg + simvastatin minimum 40 mg/day	Placebo (soybean oil) + simvastatin minimum 40 mg/day	8	ALT: 30 ± 12.7 AST: 30 ± 8.48	ALT: 0 ± 10.21 AST: 1 ± 7.81	ALT: 23 ± 8.2 AST: 26 ± 8.2	ALT: −2 ± 5.52 AST: −1 ± 5.52
15	Carrizalez et al. (2016)/Mexico	RA/PA/DB	M/F: 40 Int: 20, Con: 20	Type 2 diabetes mellitus with NPDR	Int: 58.5 ± 1.9 Con: 57.8 ± 1.9	Int: 28.2 ± 3.7 Con: 29.3 ± 0.8	CoQ10 400 mg + diabetic diet and physical activity with hypoglycemic, antihypertensive, and hyperlipidemia drugs	Placebo + diabetic diet and physical activity with hypoglycemic, antihypertensive, and hyperlipidemia drugs	24	ALT: 34.6 ± 3.1 AST: 29.8 ± 2.5	ALT: 2.1 ± 9.94 AST: −0.4 ± 7.81	ALT: 31.2 ± 1.9 AST: 28.0 ± 2.3	ALT: −1 ± 5.36 AST: −4 ± 6.35
16	Derosa et al. (2019)/Italy	RA/PA/DB	M/F: 56 Int: 29, Con: 27	Dyslipidemia patients	Int: 59.8 ± 8.3 Con: 58.3 ± 7.9	Int: NR Con: NR	Liquid CoQ10 100 mg + statins	Placebo + statins	12	ALT: 74.5 ± 52.1 AST: 68.1 ± 57.2	ALT: −21.8 ± 31.26 AST: −26.2 ± 38.59	ALT: 70.3 ± 50.2 AST: 65.9 ± 53.1	ALT: −11.5 ± 30.24 AST: −11.6 ± 31.99
17	Pek et al. (2016)/Singapore	RA/PA/DB	M/F: 40 Int: 20, Con: 20	Dyslipidemia patients	Int: 43.1 ± 11.3 Con: 49.2 ± 12.2	Int: NR Con: NR	Ubiquinol 150 mg + simvastatin (20 mg)	Placebo + simvastatin (20 mg)	12	ALT: 65.6 ± 42.3 AST: 36.8 ± 14.2 GGT: 69 ± 57.03 ALP: 78.7 ± 18.8	ALT: −9.6 ± 25.52 AST: 1.9 ± 9.04 GGT: −5 ± 35.28 ALP: −5.4 ± 11.3	ALT: 63.5 ± 16.6 AST: 36.7 ± 9.5 GGT: 54.5 ± 28.8 ALP: 79.7 ± 26.7	ALT: −11.5 ± 13.13 AST: −0.8 ± 6.94 GGT: 0 ± 18.21 ALP: −2.1 ± 16.13

Abbreviations: ALP, alkaline phosphatase; ALT, alanine transaminase; AST, aspartate aminotransferase; CHD, Coronary heart disease; GGT, gamma‐glutamyl transferase; NAFLD, nonalcoholic fatty liver disease; NPDR: Nonproliferative Diabetic Retinopathy.

^a^ Values are mean ± SD or range (for age).

^b^ Outcomes for liver enzyme concentrations are presented by common unit (IU/L).

### 3.2 Findings from the systematic review

Among 13 studies assessing the serum concentrations of ALT, five studies revealed a significant reducing effect of CoQ10 supplementation on serum ALT concentrations (Derosa et al., 2019; Emami & Bazargani‐Gilani, 2018; Farsi et al., 2016; Nevzat, 2015; Pek et al., 2016), and one study indicated a significant increase (Mabuchi et al., 2007), whereas others found no significant effect. Of 12 studies assessing the serum concentrations of AST five trials showed a significant reduction (Emami & Bazargani‐Gilani, 2018; Farhangi et al., 2014; Farsi et al., 2016; Nevzat, 2015; Pek et al., 2016), whereas others found no significant effect following CoQ10 supplementation. Of six studies that examined the effects of CoQ10 supplementation on serum GGT concentrations, four studies reported a beneficial effect (Emami & Bazargani‐Gilani, 2018; Farsi et al., 2016; Gholami et al., 2019; Nevzat, 2015), whereas others found no significant effect. Furthermore, among four studies assessing the serum concentrations of ALP, two studies revealed a significant reduction (Nevzat, 2015; Pek et al., 2016) while one study revealed a significant increase (Diaz‐Castro et al., 2020).

### 3.3 Findings from the meta‐analysis

Overall, 15 RCTs in the systematic review were included in the meta‐analysis. These trials had a total sample size of 712 individuals aged 11 years and over.

### 3.4 Effect of Q10 supplementation on the serum ALT

**FIGURE 2:**
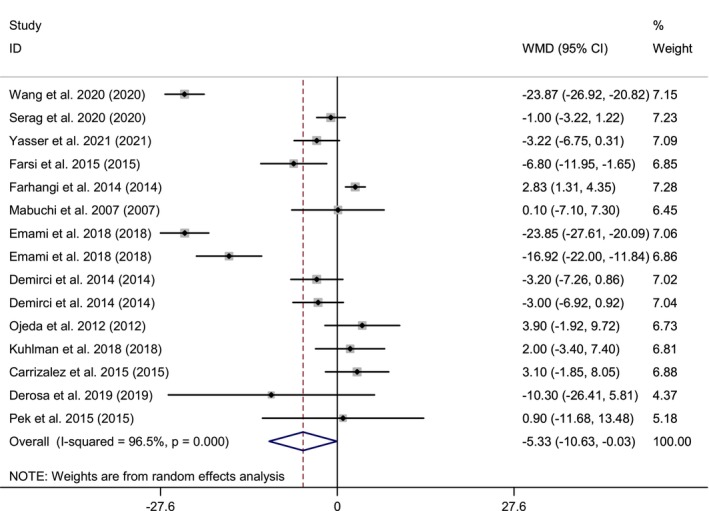
Forest plot of randomized controlled trials investigating the effects of Q10 supplementation on ALT level, expressed as mean differences between intervention and control groups. Horizontal lines represent 95% CIs. Diamonds represent pooled estimates from random‐effects analysis. ALT, alanine aminotransferase; CI, confidence interval; WMD, weighted mean difference.

In total, 13 RCTs with a total sample size of 542 subjects were included in the analysis (Derosa et al., 2019; Emami & Bazargani‐Gilani, 2018; Farhangi et al., 2014; Farsi et al., 2016; Hernández‐Ojeda et al., 2012; Kuhlman et al., 2019; Mabuchi et al., 2007; Nevzat, 2015; Pek et al., 2016; Rodríguez‐Carrizalez et al., 2016; Serag et al., 2020; Wang et al., 2020; Yasser et al., 2021). Combining 15 effect sizes from these studies indicated that CoQ10 supplementation, compared with controls, revealed a significant reduction effect of Q10 supplementation on the ALT level (weighted mean difference [WMD] = −5.33 IU/L; 95% CI: −10.63, −0.03; *p* = .04). However, significant heterogeneity was observed among the studies for this outcome (Cochran *Q* test, *p* < .001, *I* ^2^ = 96.5%) (Figure 2). Subgroup analysis by BMI, age, health condition, dose, and duration of intervention was performed and a health condition could find a possible source of heterogeneity. The effect of Q10 supplementation was more pronounced in subjects with normal weight (WMD = −13.22 IU/L; 95% CI: −24.90 to −1.53), CHD patients (WMD = −23.87 IU/L; 95% CI: −26.92 to −20.82), and athletes (WMD = −11.73 IU/L; 95% CI: −22.45 to −1.00) compared to other subgroups (Figure S1).

### 3.5 Effect of CoQ10 supplementation on the serum AST

**FIGURE 3:**
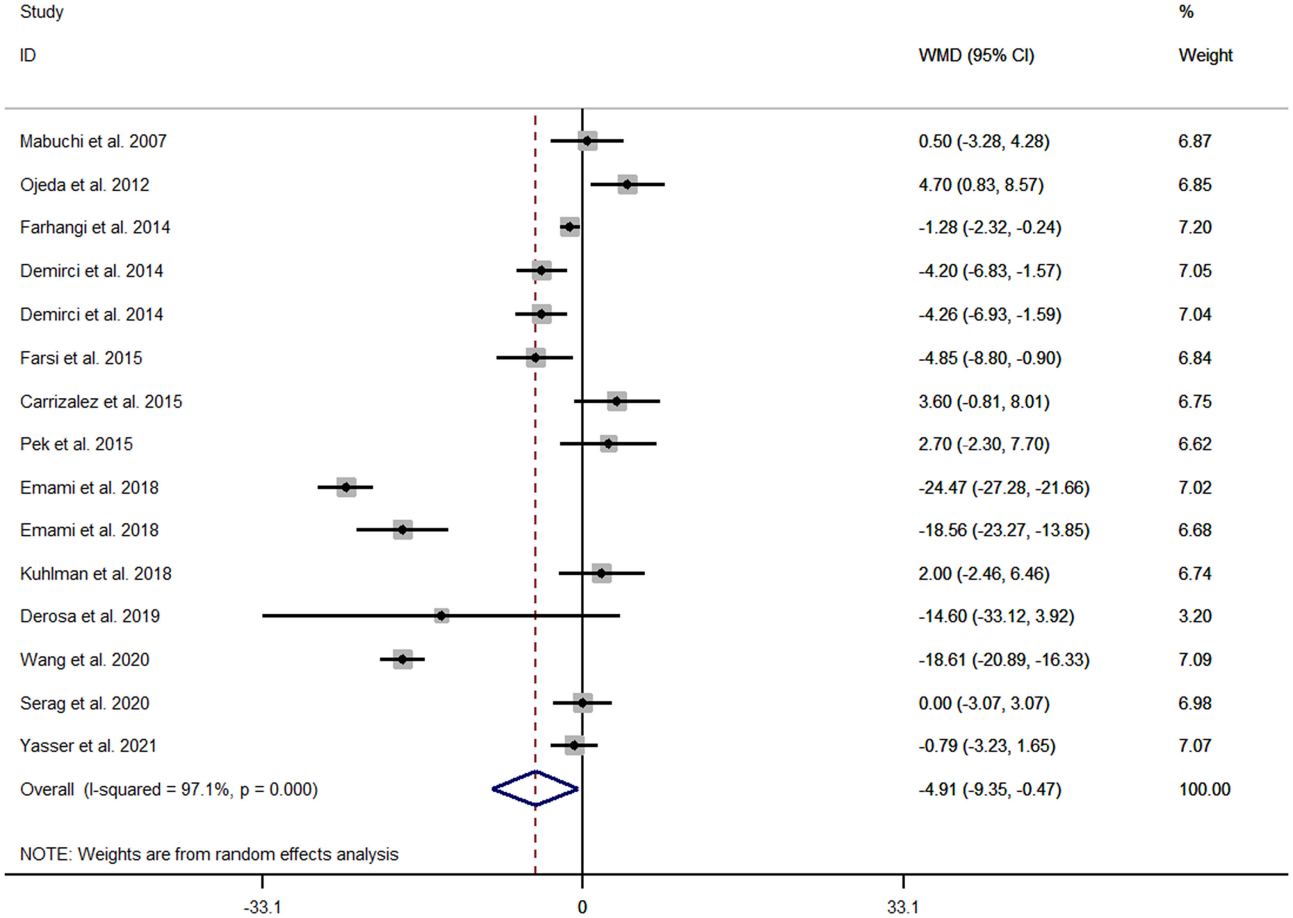
Forest plot of randomized controlled trials investigating the effects of Q10 supplementation on AST level, expressed as mean differences between intervention and control groups. Horizontal lines represent 95% CIs. Diamonds represent pooled estimates from random‐effects analysis. AST, aspartate aminotransferase; CI, confidence interval; WMD, weighted mean difference.

Combining 15 effect sizes from 13 RCTs (Derosa et al., 2019; Emami & Bazargani‐Gilani, 2018; Farhangi et al., 2014; Farsi et al., 2016; Hernández‐Ojeda et al., 2012; Kuhlman et al., 2019; Mabuchi et al., 2007; Nevzat, 2015; Pek et al., 2016; Rodríguez‐Carrizalez et al., 2016; Serag et al., 2020; Wang et al., 2020; Yasser et al., 2021), including a total sample size of 542 participants, the quantitative meta‐analysis revealed a significant effect of Q10 supplementation on the AST level (WMD = −4.91 IU/L; 95% CI: −9.35, −0.47; *p* = .03). However, significant heterogeneity was observed among the studies for this outcome (Cochran *Q* test, *p* < .001, *I* ^2^ = 97.1%) (Figure 3). Subgroup analysis by BMI, age, health condition, dose, and duration of intervention was performed. BMI categories and health conditions could find a possible source of heterogeneity. The effect of Q10 supplementation was more pronounced in subjects with normal weight (WMD = −12.24 IU/L; 95% CI: −22.37 to −2.10), in CHD patients (WMD = −18.61 IU/L; 95% CI: −20.89 to −16.33) athletes (WMD = −12.82 IU/L; 95% CI: −23.46 to −2.18) and duration ≤8 weeks (WMD = −8.43 IU/L; 95% CI: −16.05 to −0.80) compared to other subgroups (Figure S2).

### 3.6 Effect of CoQ10 supplementation on the serum GGT and ALP

**FIGURE 4:**
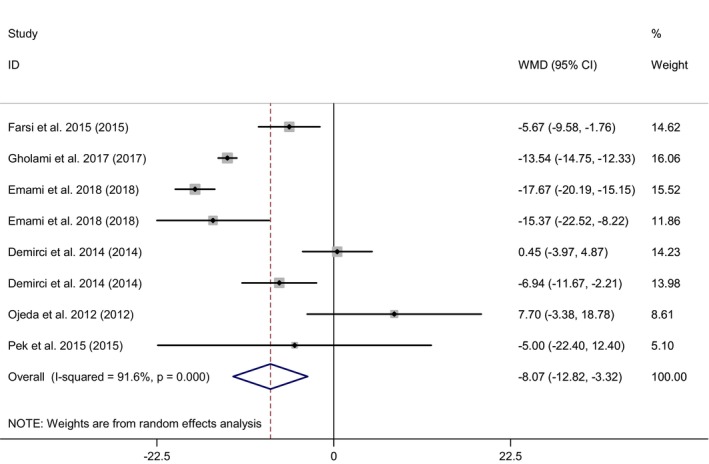
Forest plot for the effect of Q10 supplementation on GGT level, expressed as mean differences between intervention and control groups. Horizontal lines represent 95% CIs. Diamonds represent pooled estimates from random‐effects analysis. CI, confidence interval; GGT, gamma‐glutamyl transferase; WMD, weighted mean difference.

**FIGURE 5:**
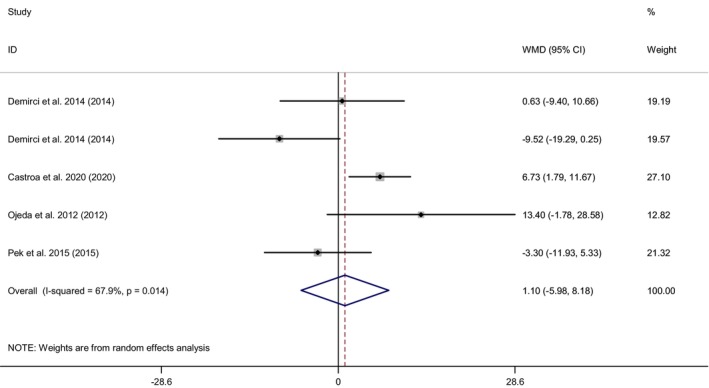
Forest plot for the effect of Q10 supplementation on ALP level, expressed as mean differences between intervention and control groups. Horizontal lines represent 95% CIs. Diamonds represent pooled estimates from random‐effects analysis. ALP, alkaline phosphatase; CI, confidence interval; WMD, weighted mean difference.

In total, eight effect sizes from six RCTs (Emami & Bazargani‐Gilani, 2018; Farsi et al., 2016; Gholami et al., 2019; Hernández‐Ojeda et al., 2012; Nevzat, 2015; Pek et al., 2016), including 228 people for the effect of Q10 supplementation on the serum GGT. Combining the effect sizes, we found a significant effect of CoQ10 supplementation on GGT (WMD = −8.07 IU/L; 95% CI: −12.82, −3.32; *p* = .001; *I* ^2^ = 91.6%) (Figure 4); while no significant changes were observed for ALP with Combining five effect sizes from four RCTs (Diaz‐Castro et al., 2020; Hernández‐Ojeda et al., 2012; Nevzat, 2015; Pek et al., 2016), including a total sample size of 199 participants (WMD = 1.10 IU/L; 95% CI: −5.98, 8.18; *p* = .76; *I* ^2^ = 67.9%) (Figure 5). Subgroup analysis by BMI, age, health condition, dose, and duration of intervention was performed. The results for GGT revealed BMI categories and age, and for ALP, BMI categories were probably heterogeneous sources. These analyses displayed that Q10 supplementation more effectively reduced GGT in subjects with normal weight (WMD = −17.42 IU/L; 95% CI: −19.79 to −15.04) ≤19 years (WMD = −17.42 IU/L; 95% CI: −19.79 to −15.04), athletes (WMD = −9.85 IU/L; 95% CI: −19.10 to −0.60), intake dose ≤200 mg Q10 (WMD = −6.46 IU/L; 95% CI: −12.87 to −0.06), and duration of intervention ≤8 weeks (WMD = −9.85 IU/L; 95% CI: −19.10 to −0.60) than controls (Figure S3). However, we found a significant increase following supplementation with CoQ10 on serum concentrations of ALP in subjects with overweight (WMD = 7.37 IU/L; 95% CI: 2.67–12.07) (Figure S4).

### 3.7 Sensitivity analysis

The leave‐one‐out method was applied to assess the influence of each study on the pooled effect size. The findings remained robust after the sequential elimination of studies (Figure S5).

### 3.8 Publication bias

The visual inspection of the funnel plot revealed no evidence of publication bias regarding the impacts of Q10 supplements on ALT, AST, GGT, and ALP. However, the results of Egger's regression test supported the insignificant publication bias for ALT (*p* = .35), AST (*p* = .60), GGT (*p* = .13), and ALP (*p* = .16) (Figure S6).

## 4 DISCUSSION

According to our knowledge, this is the first study to review the effects of CoQ10 supplementation on liver enzymes. In this study, we reviewed and summarized the results of 15 RCT studies that investigated the effects of CoQ10 supplementation on the level of liver enzymes including ALT, AST, GGT, and ALP. In the current meta‐analysis, we found that CoQ10 supplementation can exert a significant reducing effect on serum levels of ALT (5.33 IU/L), AST (4.91 IU/L), and GGT (8.07 IU/L). However, this meta‐analysis failed to find a significant effect of CoQ10 supplementation on serum levels of ALP overall.

Coenzyme Q10 is a fat‐soluble vitamin‐like quinone, known as ubiquinone, and marketed as CoQ10 (Desbats et al., 2015), and plays a crucial role in mitochondrial oxidative phosphorylation and ATP synthesis (Arenas‐Jal et al., 2020). Since CoQ10 is an essential cofactor for energy metabolism, it is also important for liver function. In addition, due to its antioxidant action (Marcoff & Thompson, 2007) can prevent cell damage, and subsequent elevation of liver enzyme levels. However, the results of some studies are different and the overall effects of CoQ10 on liver disorders and liver enzyme levels are not known with certainty.

Transaminases (especially ALT) are found mostly in hepatocytes andused as liver function markers (Jastrzębski et al., 2015; Sampson et al., 1980; Weibrecht et al., 2010; Wroblewski & Ladue, 1956a). High serum levels of aminotransferases can increase the risk of type 2 diabetes mellitus (Fraser et al., 2009), cardiovascular disease (Schindhelm et al., 2007), and mortality from vascular and nonvascular diseases (Hyeon et al., 2004). Therefore, improving liver enzyme levels, especially ALT and AST, plays an important role in preventing chronic diseases. In this meta‐analysis study, we observed a significant effect of CoQ10 supplementation in reducing serum ALT and AST levels in general. Similar to our findings in this study, in a clinical trial study by Shafieipour et al. (2018), which investigated the effects of CoQ10 supplementation on NAFLD patients, it was found that a daily intake of 60 mg of CoQ10 supplementation and also 800 IU of vitamin E in both groups for 12 weeks significantly reduced serum levels in the serum level of the liver enzymes ALT and AST. However, a meta‐analysis study (Dludla et al., 2020) in 2020 that examined the effect of CoQ10 supplementation on the clinical status of metabolic syndrome, showed that CoQ10 supplementation had no significant effect on aminotransferase levels, However, due to the main limitation of this study, which was the limitation of the number of RCT studies, and was conducted only on two studies and people with NAFLD, so it is not possible to reach general conclusions based on these results. In addition, subgroup analysis in our study showed that CoQ10 supplementation had a more pronounced reducing effect on ALT and AST levels in CHD subjects in the study of Wang et al. (2020). Patients in this study received atorvastatin, and one of the side effects of statins is the reduction of serum CoQ10 levels. Statins can lead to a decrease in the serum level of CoQ10 in several ways, including the inhibition of the HMG‐COA reductase enzyme, which is involved in the synthesis of CoQ10 in the body (Nawarskas, 2005), by reducing the transfer of CoQ10 in the plasma following a decrease in the level of LDL as a carrier of CoQ10 (Marcoff & Thompson, 2007), and also by reducing its bioavailability (Potgieter et al., 2013). Therefore, these people may have seen better effects by increasing their serum CoQ10 levels after taking CoQ10 supplements (Langsjoen & Langsjoen, 2003).

GGT is an enzyme located on the outer surface of the cell membrane, several studies have shown that GGT can be an early and sensitive marker of inflammation and oxidative stress (Emdin et al., 2005). This study observed a significant overall reduction in GGT levels with CoQ10 supplementation. In a cross‐sectional study by Onur et al., a strong relationship was shown between plasma CoQ10 status and serum GGT activity in 416 healthy participants, the strength of this relationship was gender‐dependent. It was observed more in men than in women, but a significant inverse relationship was found in all. Additionally, the second phase of this study, conducted on 53 people, showed that taking a 150 mg CoQ10 supplement for 2 weeks reduced serum GGT and GGT1 mRNA levels.

A review study using data from 9.24 million individuals showed that serum levels of GGT and ALP were positively associated with all‐cause mortality (Kunutsor et al., 2014). Unlike other liver enzymes in our study, the overall effect of CoQ10 supplementation on ALP levels was insignificant. These results may be due to the small number of RCT studies reviewed. In a clinical trial study investigating the effects of omega‐3 and CoQ10 supplementation in patients in statin‐treated patients, a significant reduction of liver enzyme levels ALP, ALT, and AST was detected in both groups in comparison to the control group. However, this effect was not significant between the two intervention groups (Tóth et al., 2017). Although most animal studies have shown the beneficial effects of CoQ10 supplementation in reducing ALP levels (Ahmadvand & Ghasemi‐Dehnoo, 2014; Esfahani et al., 2013), results in human studies are contradictory. The study conducted by Diaz‐Castro et al. (2020) on 100 male firefighters showed that plasma ALP levels were significantly increased following 8 weeks of 200 mg/day CoQ10 supplementation. Indeed, in the absence of liver disease, serum ALP, one of the key markers of bone metabolism, indicates osteoblastic activity (Holdsworth et al., 2019; Neve et al., 2011). Previous studies have also found that CoQ10 increases the expression of osteogenic markers such as bone ALP (BALP) and has antiosteoporotic effects (Moon et al., 2013; Zheng et al., 2018). Therefore, the conflicting results in the studies can be due to different reasons, including the difference in the type of ALP or the baseline serum levels of ALP in different individuals, the dose of CoQ10 received, the sample size, or the length of the intervention. Although subgroup analyses were conducted for intervention duration, age, health and disease status, dietary supplement dosage, and BMI, the results are unreliable due to the high level of subgroup heterogeneity. Moreover, the number of subgroup studies was sometimes small, especially for ALP. Various risk factors, such as drugs, obesity, and metabolic diseases, reduce levels of antioxidants such as glutathione and superoxide dismutase in the liver and increase the production of free radicals such as ROS and RNS (Bournat & Brown, 2010; Li et al., 2015; McClain et al., 1999). Oxidative stress causes hepatocyte damage and destruction, leading to increased levels of liver enzymes in plasma. Oxidative stress in the liver can also lead to the disruption of mitochondrial beta‐oxidation. This is one of the major factors in the pathogenesis of NAFLD, leading to the accumulation of fatty acids in hepatocytes, disease progression, and subsequent increased levels of Liver enzymes in plasma (Fernández‐Sánchez et al., 2011; Li et al., 2015; Muriel & Gordillo, 2016). CoQ10, as a part of the mitochondrial respiratory chain, prevents the endogenous production of ROS in mitochondria, which is also associated with a decrease in MDA levels and an increase in SOD activity (Jing et al., 2015; Karajibani et al., 2009), thus acting as a powerful antioxidant in the body and protecting against hepatocyte damage. CoQ10 also increases the PPAR‐ɑ expression and fatty acid oxidation, therefore having a positive effect on lipid metabolism and improving dyslipidemia (Moazen et al., 2015). In addition, Coq10 suppresses the synthesis and accumulation of lipids and triglycerides in the liver by reducing the mRNA expression of lipogenic enzymes such as fatty acid synthase (FAS) and acetyl carboxylase (ACC1), and the glycerogenic enzyme phosphoenol pyruvate carboxylase (PEPCK), thereby prevents liver cell damage and elevated liver enzyme levels (Alam & Rahman, 2014). Elevated aminotransferase level is also one of the indicators used to assess muscle damage. The oxidative stress generated in muscle cells after intense and prolonged physical activity is associated with tissue damage. When the muscle is damaged, AST and ALT are released from the muscle, resulting in transient increases in liver function tests (Halonen & Konttinen, 1962). Studies have shown that due to the important role of coenzyme Q10 in mitochondria and as an antioxidant, CoQ10 supplementation can reduce oxidative stress following exercise and thus reduce muscle and liver cell damage and the level of liver enzymes. In addition, the beneficial effects of CoQ10 on liver enzymes may be due to its beneficial effects on inflammation. Inflammatory factors are involved in liver dysfunction and elevated liver enzyme levels (Alam & Rahman, 2014). Therefore, the anti‐inflammatory effects of CoQ10 are effective in improving liver function and reducing liver enzyme levels. CoQ10 may play a potential role in reducing the production of inflammatory cytokines such as TNF‐α by inhibiting NF‐κB and hs‐CRP gene expression and reducing ICAM‐1 activation (Olivieri et al., 2013; Schmelzer et al., 2008). A meta‐analysis study found that CoQ10 supplementation could increase adiponectin levels by decreasing ROS production (Dludla et al., 2020). Adiponectin is an anti‐inflammatory cytokine that has anti‐TNF‐α effects, increases insulin sensitivity, and has protective effects on hepatocytes and NAFLD (Farsi et al., 2019; Jorat et al., 2019; Zhai et al., 2017). According to the role of CoQ10 in the body described, CoQ10 supplementation is expected to have beneficial effects on liver and hepatocyte function, thereby reducing serum liver enzymes.

The main strength of our study is that it is the first systematic review and meta‐analysis study to examine and summarize the results of clinical trial studies related to the effects of CoQ10 supplementation on the level of liver enzymes. Furthermore, it was determined that there was no publication bias in this meta‐analysis study by using Egger's test. However, some limitations should be considered in this study. First, in most of the entered clinical studies, measurement of liver enzymes was a secondary outcome, and RCTs were conducted to determine the safety of CoQ10 supplementation in people. Moreover, the bioavailability of CoQ10 and its plasma levels after supplementation has not been investigated in some studies, so the amount of CoQ10 available in the blood after ingestion is not clearly defined. In addition, most subjects included in the RCTs had normal liver enzyme levels. On the other hand, in some studies, people in the control group did not receive a placebo, which may have affected the results of the studies. Another limitation of this study is the high heterogeneity of the findings. This may be due to the different health conditions of the participants and their medications. In addition, there are some confounding factors, such as small sample size or short intervention period in some clinical studies.

Overall, the results of this systematic review and meta‐analysis suggest that CoQ10 supplementation can reduce the serum concentration of liver enzymes ALT, AST, and GGT, commonly used as liver function biomarkers. However, this significant effect was not observed for ALP in general. Therefore, coenzyme Q10 may be effective in protecting body cells, especially liver cells, and reducing levels of main liver enzymes. However, the mechanism of the effect is not known, and more RCTs with better designs and larger sample sizes are needed to confirm the results of our study.

## AUTHOR CONTRIBUTIONS

**Mohadeseh Soleimani Damaneh:** Conceptualization (equal); data curation (equal); investigation (equal); methodology (equal); resources (equal); software (equal); validation (equal); visualization (equal); writing – original draft (equal); writing – review and editing (equal). **Somaye Fatahi:** Data curation (equal); methodology (equal); resources (equal); software (equal); validation (equal); writing – review and editing (equal). **Naheed Aryaeian:** Methodology (equal); project administration (equal); supervision (equal); validation (equal). **Hossein Bavi Behbahani:** Investigation (equal); software (equal); writing – original draft (equal); writing – review and editing (equal).

## CONFLICT OF INTEREST STATEMENT

Authors declared no personal or financial conflicts of interest.

## Supporting information


Figures S1–S6.

Table S1.

## Data Availability

Data will be made available on request.
