# Supplementary material for: The effect of coenzyme Q10 supplementation on liver enzymes: A systematic review and meta‐analysis of randomized clinical trials
Source: Food Sci Nutr. 2023 Jun 7;11(9):4912–25. doi: 10.1002/fsn3.3478 (PMC10494615; doi:10.1002/fsn3.3478)
Supplement: Supplementary file 1 — Figures S1–S6. [file FSN3-11-4912-s002.docx]

**Supplementary file:**

| A)   | B)   |
| --- | --- |
| C)   | D)  |
| E)   | F) |
| **Supplementary figure1.** Forest plot of randomized controlled trials investigating the effects of Q10 supplementation on ALT level based on A) BMI, B) Age, C) Health condition, D) Dose and , E) Duration of intervention | |

| A)   | B)   |
| --- | --- |
| C)   | D)   |
| E)   |  |
| **Supplementary figure2.** Forest plot of randomized controlled trials investigating the effects of Q10 supplementation on AST level based on A) BMI, B) Age, C) Health condition, D) Dose and, E) Duration of intervention | |

| A)   | B)   |
| --- | --- |
| C)   | D)   |
| E)   |  |
| **Supplementary figure3.** Forest plot of randomized controlled trials investigating the effects of Q10 supplementation on GGT level based on A) BMI, B) Age, C)Health condition, D) Dose and, E) Duration of intervention | |

| A)   | B)   |
| --- | --- |
| C)   | D)   |
| E)   |  |
| **Supplementary figure4.** Forest plot of randomized controlled trials investigating the effects of Q10 supplementation on ALP level based on A) BMI, B) Age, C) Health condition, D) Dose and, E) Duration of intervention | |

| A)   | B)   |
| --- | --- |
| C)   | D)   |
| **Supplementary figure5.** The leave-one-out method on the pooled effect size for A) ALT, B) AST, C) GGT, and D) ALP | |

| A)   | B)   |
| --- | --- |
| C)   | D)   |
| **Supplementary Figure 6.** Funnel plot of the weighted mean difference (WMD) versus the standard error (s.e) for A) ALT, B) AST, C) GGT, and D) ALP. | |
