# Supplementary material for: The effect of coenzyme Q10 supplementation on liver enzymes: A systematic review and meta‐analysis of randomized clinical trials
Source: Food Sci Nutr. 2023 Jun 7;11(9):4912–25. doi: 10.1002/fsn3.3478 (PMC10494615; doi:10.1002/fsn3.3478)
Supplement: Supplementary file 2 — Table S1. [file FSN3-11-4912-s001.docx]

**Supplemental Table 1**: Results of risk of bias assessment for randomized clinical trials included in the current meta-analysis on the effects of CoQ10 supplementation on Liver enzeymes^1^

|  | Study | Random Sequence Generation | Allocation concealment | Reporting bias | Other sources of bias | Performance bias | Detection bias | Attrition bias |
| --- | --- | --- | --- | --- | --- | --- | --- | --- |
| 1 | Farsi et al. 2015 | L | L | L | L | L | U | L |
| 2 | Farhangi et al. 2014 | L | U | L | L | L | U | L |
| 3 | Wang et al. 2020 | L | U | L | L | U | U | L |
| 4 | Serag et al. 2020 | L | L | L | H | U | H | H |
| 5 | Yasser et al. 2021 | L | H | L | H | U | U | H |
| 6 | Mabuchi et al. 2007 | L | U | L | L | L | L | L |
| 7 | Gholami et al. 2017 | L | U | H | L | L | H | U |
| 8 | Emami et al. 2018 | L | U | L | L | U | H | L |
| 9 | Demirci et al. 2014 | L | U | L | H | U | L | H |
| 10 | Castro et al. 2020 | L | U | H | H | L | H | L |
| 11 | Ojeda et al. 2012 | L | U | L | H | L | H | L |
| 12 | Kuhlman et al. 2018 | L | U | L | L | L | H | L |
| 13 | Carrizalez et al. 2015 | L | U | L | L | L | H | L |
| 14 | Derosa et al. 2019 | L | L | L | L | L | U | L |
| 15 | Pek et al. 2015 | L | U | L | H | L | H | L |

^1^Each study was assessed for risk of bias using the Cochrane Risk of Bias Assessment tool (Ref. 24). Domains of assessment included random sequence generation, allocation concealment, reporting bias, performance bias, detection bias, attrition bias, and, other sources of bias. Each domain was scored as “high risk” if it contained methodological flaws that may have affected the results, “low risk” if the flaw was deemed inconsequential, and “unclear risk” if the information was insufficient to determine. If a study got “low risk” for all domains, it considered as a high quality study with totally low risk of bias.
